# Response to “Conceptual and Disclosure Considerations in Applying Neuroaesthetics to Aesthetic Medicine”

**DOI:** 10.1111/jocd.71148

**Published:** 2026-08-16

**Authors:** Steven Dayan

**Affiliations:** ^1^ University of Illinois Chicago Illinois USA

To the Editor,

We appreciate the interesting commentary by Harris and Michon regarding our recent article introducing a neuroaesthetic framework for facial aesthetic medicine [1, 2]. Scientific progress depends on rigorous debate, and we welcome the opportunity to further clarify the intent of our work.

Importantly, there appears to be considerably more agreement than disagreement between our perspectives. Harris and Michon acknowledge that observer perception is neurally mediated, that naturalness and facial perception remain underexplored within aesthetic medicine, and that empirical investigation of these concepts is warranted. These points reflect the motivation behind our article. Our objective was not to declare neuroaesthetics a completed scientific theory within aesthetic medicine, but rather to propose a conceptual framework that integrates established findings from neuroscience, evolutionary psychology, and aesthetic medicine into a coherent research agenda.

The correspondence from Harris and MIchon repeatedly evaluates our article as though it were intended to function as a systematic review or definitive evidentiary statement. It was neither and never claimed as such. We intentionally described its purpose as conceptual rather than quantitative. Narrative scholarship has long served an important role in medicine by organizing observations across disciplines, identifying patterns, and generating hypotheses. It complements, rather than replaces, systematic reviews and randomized trials.

Throughout the manuscript we intentionally distinguished established evidence from emerging observations. Where evidence was preliminary, we explicitly stated that it should be interpreted cautiously and that further investigation was necessary. The discussion of visually impaired individuals detecting facial attractiveness, for example, was presented not as proof of a new mechanism but as an invitation to broaden scientific inquiry. Likewise, our discussion of facial feedback, predictive coding, and other neuroscientific concepts was offered as a plausible framework through which future clinical studies might be designed rather than as conclusive evidence.

The history of scientific progress suggests that conceptual models frequently precede experimental validation. New fields rarely emerge from a single trial. Rather, they develop when observations from multiple disciplines are synthesized into hypotheses that can be tested, refined, challenged, or rejected. This process has characterized advances throughout medicine and science. Advancement in medicine should not be evaluated only by the questions it answers, but perhaps even more so by the quality of the questions it encourages.

This approach has characterized much of our previous academic work. Over the past two decades, we have published numerous conceptual articles introducing ideas that initially extended beyond conventional thinking, including observer‐reported outcomes, patient‐reported outcomes, first‐impression research, subliminal facial change, the psychology of attractiveness, perception drift, aesthetic bias, special theory of relativity in attractiveness, illusion of time, truth and aesthetic medicine, mind mood and aesthetics, decoding GLP's and quite a few other interdisciplinary models integrating behavioral science with facial aesthetics. Each was offered not as immutable truth but as a framework intended to stimulate broader thinking, spirited discussion and encourage rigorous investigation. Some concepts have subsequently gained wide acceptance, while others continue to evolve through ongoing scientific inquiry. We view neuroaesthetics in aesthetic medicine as part of that same academic tradition.

We also wish to address the comments regarding disclosure. Our manuscript disclosed our professional relationships in accordance with journal policy. We strongly support transparent disclosure because readers deserve the opportunity to evaluate potential competing interests. At the same time, we believe scientific arguments should ultimately be judged on their evidentiary foundation rather than presumed commercial associations.

For this reason, we were somewhat surprised by the implications from an author who serves as an editor of the journal that accepted our paper as well as a consultant to the skin care industry, that our article functioned as a promotional extension of a commercial skincare program. The manuscript neither discusses XOMD, recommends any proprietary product, introduces the term neurocosmetics, nor proposes skincare as the central application of neuroaesthetic principles. Instead, the discussion centers on facial identity, observer perception, fillers, neuromodulators, surgery, movement, emotional expression, and the neuroscience underlying how faces are perceived. The conceptual framework presented is substantially broader than any individual product category and was intended to stimulate investigation across the entirety of facial aesthetic medicine rather than support a commercial intervention.

Scientific disagreement is both healthy and necessary. However, it is equally important that conceptual scholarship be evaluated within the context in which it was written. Our article did not claim neuroaesthetics as fully validated within aesthetic medicine. It argued that sufficient evidence now exists across multiple scientific disciplines to justify incorporating neuroaesthetic principles into future research, outcome assessment, and clinical thinking. We continue to believe this represents a worthwhile direction for our specialty and hope the discussion initiated by our article inspires the next generation of conceptual thinkers to pursue the prospective investigations that will ultimately determine the clinical significance of these ideas.

Scientific progress has always begun with a hypothesis bold enough to challenge conventional wisdom and disciplined enough to withstand all levels of scrutiny.

## Disclosure

Consultant, Principle Investigator, and/or Speaker for ABBVIE, Revance, Teoxane, Evolus, Veradermics, Galderma. Co‐Founder XOMD.

## Conflicts of Interest

The author declares no conflicts of interest.

## Data Availability

The data that support the findings of this study are available from the corresponding author upon reasonable request.

## References

[1] S. Harris and A. Michon , “Conceptual and Disclosure Considerations in Applying Neuroaesthetics to Aesthetic Medicine,” Journal of Cosmetic Dermatology 25, no. 7 (2026): e71061.

[2] S. Dayan and S. Fabi , “Neuroaesthetics: Evolutionary Thinking in Facial Aesthetic Medicine,” Journal of Cosmetic Dermatology 25, no. 5 (2026): e70885. 42108601 10.1111/jocd.70885 PMC13158439

